# Evaluation of Neuromuscular Morphometry of the Vaginal Wall Using Protein Gene Product 9.5 (Pgp 9.5) and Smooth Muscle α-Actin (Sma) in Patients with Posterior Vaginal Wall Prolapse

**DOI:** 10.3390/medicina60050816

**Published:** 2024-05-16

**Authors:** Mustafa Çetin, Güven Güney, Özer Birge, Emine Arslan, Burcu Timur, Hakan Timur

**Affiliations:** 1 Department of Gynecology and Obstetrics, Ordu Training and Research Hospital, 52200 Ordu, Turkey; mustafacetin87@gmail.com (M.Ç.); drburccu@gmail.com (B.T.); 2 Department of Gynecology and Obstetrics, Hitit Üniversitesi, 19030 Çorum Merkez, Turkey; dr.guvenguney61@gmail.com (G.G.); ekaranfildr@gmail.com (E.A.); 3 Department of Obstetric and Gynecology, Maternite de I’Amitie Turqui-Niger Hospital, Niamey 920271, Niger; ozbirge@gmail.com; 4 Department of Gynecology and Obstetrics, Ordu University, 52200 Ordu, Turkey

**Keywords:** posterior vaginal wall prolapse, Protein Gene Product 9.5, α smooth muscle actin

## Abstract

*Background and Objectives*: This study aims to compare the neuromuscular structure of the vagina in women with posterior vaginal wall prolapse with the neuromuscular structure of the vagina in women without prolapse, to determine the difference, and to demonstrate the role of neuromuscular structure in the physiopathology of prolapse. *Materials and Methods*: In this prospective study, women aged between 40 and 75 years who had not undergone any vaginal surgery and had not undergone any abdominal prolapse surgery were included. Thirty-one women diagnosed with rectocele on examination were included in the study group. Thirty-one patients who underwent vaginal intervention and hysterectomy for reasons other than rectocele (colposcopy, conization, etc.) without anterior or posterior wall prolapse were included in the control group. Biopsy material was obtained from the epithelium of the posterior wall of the vagina, including the fascia that fits the Ap point. Immunohistochemical staining with Protein Gene Product 9.5 and smooth muscle α-actin was performed in the pathology laboratory. The epithelial thickness measurement and smooth muscle density parameters obtained with these immunohistochemical stainings were compared between the two groups. The collected data were analyzed using the SPSS 23 package program. *p* values less than 0.05 were considered statistically significant. *Results*: In the control group, muscle thickness and the number of nerves per mm^2^ of fascia were statistically significantly higher than in the study group (*p* < 0.05). *Conclusions*: We found that smooth muscle tissue and the number of nerves per mm^2^ of fascia were decreased in posterior vaginal wall prolapse compared to the general population. Based on the correlation coefficients, age was the parameter that most affected the degree of prolapse, followed by parity, number of live births, and number of vaginal deliveries.

## 1. Introduction

Pelvic organ prolapse (POP) is a common condition in which the pelvic organs herniate into or out of the vaginal walls. Many women with herniation have symptoms affecting their daily activities and sexual life. The presence of POP has negative effects on body perception and sexuality [1].

As with pelvic organ prolapse, rectocele is the result of people standing on two legs [2]. As the symptoms and conditions of pelvic floor dysfunction span a broad spectrum of disciplines, determining the overall incidence of the amalgam of disorders is difficult. By the age of 80, about 11% of women will have one or more surgical interventions for urinary incontinence or pelvic organ prolapse [3]. The progressive weakening of the pelvic floor, a natural consequence of aging, and the joint effect of birth-related trauma lead to the chronic deterioration of the rectovaginal septum, resulting in rectocele [4]. In most patients, multiparity and chronic causes of increased intra-abdominal pressure are prominent in the etiology [5]. Rectocele is a relatively common disease. Its prevalence increases with age, history of constipation, multiple vaginal deliveries, and episiotomies. However, rectocele and clinical problems due to rectocele can also be encountered in female patients who have never been pregnant [6,7]. The age range of clinical manifestations of rectocele is usually between the fourth and fifth decades. The clinical symptoms of the patients are not compatible with the size of the detected rectocele [8]. Nevertheless, rectoceles over 2 cm in size are symptomatic [9]. The first symptoms encountered may be mild complaints such as constipation and vaginal fullness [10].

The endopelvic fascia, extending into the inner layers of the posterior vaginal wall, is the most crucial fascia within the rectovaginal septum. The rectovaginal fascia and the side walls of the paracolpium support the posterior wall of the vagina. According to DeLancey, the rectovaginal fascia is a fibromuscular structure starting from the peritoneum and extending to the perineal body, and it is more prominent in the lateral parts than in the medial parts [11]. This ensures that the movements of the vagina and rectum are independent of each other.

The rectovaginal fascia provides passive support to the visceral organs and pelvic floor. The rectovaginal fascia is formed by collagen, fibroblasts, smooth muscle fibers, elastin, neurovascular, and fibrovascular fibers. The rectovaginal fascia secures the endopelvic fascia, cervix, and vagina to the pelvis on either side of the pelvis. The endopelvic fascia is the structure that surrounds the pelvic organs and provides loose attachment to the pelvic bones and pelvic diaphragm. The endopelvic fascia contains smooth muscle fibers, vascular nerve bundle, adipose tissue, collagen, and elastin, and it is the most essential support structure used to stabilize the uterus [12,13,14].

Prolapse of the posterior wall of the vagina is caused by weakness in the endopelvic fascia [15]. Rectocele occurs due to stretching and rupture of the rectovaginal fascia from the attachment points due to the expansion of the vaginal wall during labor. Furthermore, systemic diseases and hereditary connective tissue diseases may also cause rectocele [16]. Studies on posterior vaginal wall prolapse are usually retrospective. There are very few case–control studies on this subject. This study aims to clinically evaluate underlying factors in the etiology of posterior vaginal wall prolapse, which leads to rectal and sexual dysfunction, affecting quality of life. The aim of this study is to evaluate and compare this neuromuscular structure in women with posterior vaginal wall prolapse with the neuromuscular structure of women in the general population. It was intended to determine epithelial thickness, collagen tissue properties and the amount and characteristic of staining, and the extent of change with age in the evaluation of the formed tissue in the age range in which this condition is observed.

The effect of the thickness and morphometry of the rectovaginal fascia on posterior vaginal wall prolapse treated with various surgical interventions was evaluated. In this study, we evaluated the role of the rectovaginal fascia, and its structure, thickness, and smooth muscle density in the etiology of rectocele. Studies evaluating the neuromuscular morphometry of the anterior pelvic fascia are available in the literature [17,18]. Biopsies of the anterior vaginal wall during surgery in women with prolapse showed the altered expression of smooth muscle proteins and decreased smooth muscle fraction [19,20].

It has also been observed in some skeletal muscle tumors, myofibroblasts, and myoepithelial cells in pathological tissues that the smooth muscle actin (SMA) contingent can be controlled during translation and transcription [21]. It is considered that myofibroblasts involved in tissue damage and wound healing originate from pericytes, vascular smooth muscles, and perivascular fibroblasts and are transported to the wound [22]. Unlike the others, α SMA, which is one of the SMA types, is generally detected in cells of smooth-muscle origin. It has been reported that the expression of smooth muscle proteins is differentiated at various levels, and that there is a decrease in smooth muscle fraction in women with prolapse biopsied from the anterior vaginal wall [19,20].

Protein Gene Product 9.5 (PGP 9.5), also known as ubiquitin carboxyl-terminal hydrolase-1, is a 27 kDa protein first isolated from whole-brain extracts [23]. American guidelines consider anti-Protein Gene Product 9.5 immunohistochemistry to be the gold standard in the evaluation of distal symmetric polyneuropathy and the determination of intraepidermal nerve fiber density by skin biopsy [24]. Similarly, European guidelines conclude that distal leg skin biopsy measuring the amount of intraepidermal nerve fiber density is a reliable and effective technique to assess the diagnosis of fine fiber neuropathy [25]. Changes in vaginal mucosal innervation have previously been described in patients with vulvar–vestibulitis pain syndrome [26] and stress urinary incontinence [27] using the PGP-9.5 neuronal marker. Further studies are needed to neurochemically characterize the nerve fibers of the rectovaginal wall in patients with rectocele.

The aim of this study, “Evaluation of neuromuscular morphometry of the vaginal wall using PGP-9.5 and SMA in women with posterior vaginal wall prolapse”, is to compare this neuromuscular structure in women with posterior vaginal wall prolapse with the neuromuscular structure of women in the general population and perform an evaluation.

## 2. Materials and Methods

A total of 62 patients admitted to the Gynecology and Obstetrics Clinic of Hitit University Training and Research Hospital between December 2019 and June 2020 were included in the study. Patients aged 40–75 years were included in both groups. In the study, the subjects were divided into two groups. Patients operated on for prolapse were included in the study group. Patients who did not have prolapse and who were undergoing vaginal intervention for other gynecological reasons constituted the control group. Both groups were planned to contain 31 patients each. The informed consent form was read to all patients who participated in the study, and the patients who agreed to sign the form were included in the study. The first group included women aged between 40 and 75 years, who had not undergone any vaginal surgery, had not undergone any abdominal uterine suspension surgery, had posterior wall prolapse, and for whom surgery was planned. The second group included women between the ages of 40 and 75 who had not undergone any vaginal surgery, had not undergone any abdominal uterine suspension surgery, did not have posterior vaginal wall prolapse, and who were scheduled to undergo vaginal intervention for other reasons. Patients whose age was not suitable for the specified group, who had previously undergone vaginal surgery, who had undergone abdominal surgery and a uterine suspension surgery, who were not suitable for the specified examination conditions, and who had undergone rectocele surgery, experienced urinary incontinence, or undergone bladder surgery were not included in the study.

Of the patients who agreed to participate in the study, reproductive information such as age, height, weight, smoking, previous operations, concomitant chronic diseases, gravida, parity, abortus, number of living children, number of vaginal deliveries, number of C-sections (CSs), date of last menstrual period, and duration of menopause presented in the patient/healthy case form was recorded both to identify the patient and to examine them in the study. If prolapse was present in all patients, its grade was staged according to POP-Q classification and recorded.

In both groups, samples approximately 2–6 mm thick and 5–9 mm wide were taken from 3 cm proximal to the vagina at the Ap point according to POPQ classification during the interventional procedure. The samples taken in the pathology clinic were fixed in 10% formaldehyde for 6–8 h, and a macroscopic examination was performed. All samples sent for macroscopic examination were sliced into 5 mm thick sections and cassette-taped from surface to depth. The obtained cassettes were then subjected to 16 h alcohol monitoring in a fully automatic tissue tracking device. Three slides and 4-micron-thick sections were obtained from the paraffin-embedded control and case groups for routine Hematoxylin-Eosin staining. The sections obtained were evaluated under a light microscope. Blocks containing the most muscle tissue and peripheral nerve sections were selected, and immunohistochemical studies were performed. The findings were evaluated by an expert pathologist in a completely blinded manner.

**Figure 1:**
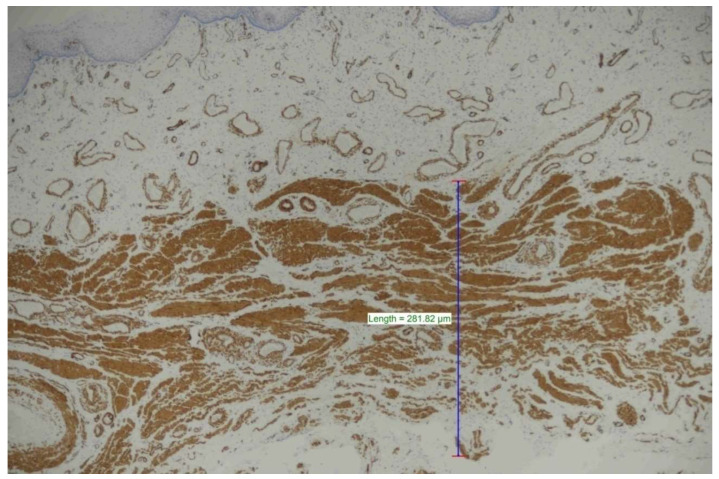
Measurement made with the NIS elements program from the place where muscle thickness was greatest (SMA ×40).

Smooth muscle actin (SMA) (clone monoclonal Mouse anti-human Smooth Muscle Actin clone 1A4) was studied in a DAKO (Omnis, EnVisionTM FLEX, High pH Code GV800) fully automatic immunohistochemistry staining device for the evaluation of muscle thickness in the samples. The stained preparations obtained were measured for each case using a software program (NIS element) by determining the thickest part of the muscle under a Nikon Eclipse Ni light microscope (Figure 1).

**Figure 2:**
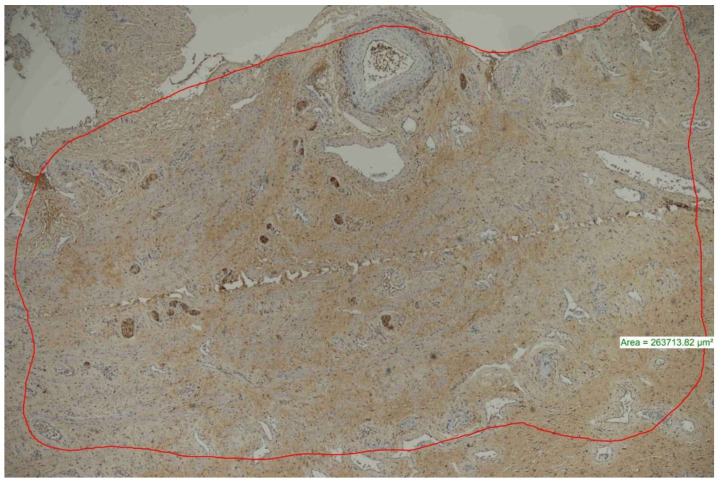
The area with the highest number of nerve sections was measured and proportioned to mm^2^ (PGP 9.5 ×40).

To evaluate the number of peripheral nerves in the samples, a PGP 9.5 (clone Polyclonal Rabbit Anti-PGP 9.5 Code No./Code/Code-Nr. Z 5116) DAKO (Omnis, EnVisionTM FLEX, High pH Code GV800) fully automatic immunohistochemistry staining device was used. The stained preparations were examined under a Nikon Eclipse Ni light microscope, and the number of peripheral nerves per mm^2^ was determined using a software program (NIS element) (Figure 2).

### 2.1. Statistical Analysis

The SPSS version 23 package program was used for the statistics of this study. The following were analyzed: the distribution of age, height, weight, BMI, smoking status, operation, and chronic diseases of the control and study groups and the results of the difference analysis; the mean values of some parameters related to delivery in the study and control groups and the results of the difference analysis between the groups; distribution of labor parameters that were significant between the study and control groups; the mean values of prolapse, POPQ Ap, POPQ Bp, muscle thickness, and number of nerves per mm^2^ of fascia in the study and control groups, and the results of difference analysis; the means of prolapse, POPQ Ap, POPQ Bp, muscle thickness, and number of nerves per mm^2^ in fascia in the study and control groups; Pearson’s correlation analysis results for the relationship between prolapse, POPQ Ap, POPQ Bp, and muscle thickness, with number of nerves per mm^2^ in fascia in the study group; the results of the correlation analysis for the relationship between the degree of prolapse with some demographic and obstetric characteristics of the patients in the study group; Spearman’s rho correlation analysis results for the relationship between POPQ Ap and POPQ Bp parameters with some demographic and birth characteristics of the patients in the study group; Spearman’s rho correlation analysis results for the relationship between the parameters of muscle thickness and number of nerves per mm^2^ in fascia with some demographic and birth characteristics of the patients in the study group. *p* values < 0.05 were considered significant.

### 2.2. Ethical Statement

Before the study, approval was obtained from the Hitit University Faculty of Medicine Clinical Research Ethics Committee with the number 116, dated 12 November 2019. The clinical trial registration number of the study is NCT06363838.

## 3. Results

This study aims to demonstrate the structural changes in women with rectoceles compared to the general population using SMA and PGP 9.5 stains.

In the control group of 31 patients, 25 patients underwent hysterectomy + bilateral salpingo-oophorectomy and constituted the majority. The rest of the control group consisted of three patients with probe curettage, one with hysterectomy, one with hysterectomy + right unilateral salpingo-oophorectomy, and one with hysterectomy + bilateral salpingo-oophorectomy + left ovarian cyst excision. Of the 31 patients in the study group, 11 patients underwent vaginal hysterectomy + anterior–posterior colporrhaphy and 9 patients underwent anterior–posterior colporrhaphy. Two patients each underwent anterior colporrhaphy, total abdominal hysterectomy + bilateral salpingo-oophorectomy, and anterior–posterior colporrhaphy + uterosacral sacrocolpopexy surgeries; one patient each underwent vaginal hysterectomy, total abdominal hysterectomy + posterior colporrhaphy, posterior colporrhaphy, perinoplasty, and bilateral tubal ligation + transobturator tape + posterior colporrhaphy.

The mean age of the control group (49.87 ± 5.35) was statistically significantly lower than the mean age of the study group (61.13 ± 8.74) (*p* < 0.05). Mean height was higher in the control group and mean weight and BMI were higher in the study group; however, the differences between the groups were not statistically significant (*p* > 0.05). Overall, 3.2% of the control group and 10.0% of the study group reported smoking. Smoking did not show a statistically significant difference between the groups (*p* > 0.05). Overall, 45.2% of the control group and 61.3% of the study group had a history of surgery. The differences between the distribution of the operation history of both groups were not statistically significant (*p* > 0.05). Overall, 48.4% of the control group and 71.0% of the study group had a history of chronic disease, and again, the difference in the distribution of chronic disease history between the groups was not statistically significant (*p* > 0.05).

The mean gravida, parity, abortus, number of living children, number of vaginal deliveries, and duration of menopause were higher in the study group compared to the control group. The number of CSs was higher in the control group than in the study group. Based on the results of the difference analysis, the differences between the groups in terms of gravida, parity, number of living children, number of normal vaginal births, and duration of menopause were statistically significant (*p* < 0.05). The differences in the mean number of abortus and CSs between the groups were not statistically significant (*p* > 0.05). Means and ranges of change in gravida, parity, abortus, live births, number of normal vaginal births (NSDs), and duration of menopause were higher in the study group than in the control group.

The mean POPQ Ap and POPQ Bp were significantly higher in the control group, while muscle thickness and number of nerves per mm^2^ of fascia were significantly higher in the study group (*p* < 0.05). These distributions are shown in Table 1. As shown, POPQ Ap and POPQ Bp averages and ranges of change were higher in the study group. There was a more stable distribution with both lower averages and a smaller range of variation in the control group. The opposite was true for muscle thickness and the number of nerves per mm^2^ of fascia.

The results of Pearson’s correlation analysis for the relationship between prolapse, POPQ Ap, POPQ Bp, and muscle thickness with number of nerves per mm^2^ in fascia in the study group are given in Table 2.

Based on the correlation analysis results, there was a statistically significant and positive correlation between the degree of prolapse and POPQ Ap (r = 0.915; *p* < 0.01) and POPQ Bp (r = 0.912; *p* < 0.01). The correlation coefficient showed that the parameter that most affected the degree of prolapse was POPQ Ap. There was also a statistically significant and positive correlation between muscle thickness and the number of nerves per mm^2^ in the fascia (r = 0.618; *p* < 0.01). There was no statistically significant correlation between the degree of prolapse, POPQ Ap and POPQ Bp, and muscle thickness with the number of nerves per mm^2^ of fascia (*p* > 0.05).

The results of the correlation analysis for the relationship between the degree of prolapse with some demographic and obstetric characteristics of the patients in the study group are given in Table 3. Based on the results of correlation analysis, there were statistically significant and positive correlations between the degree of prolapse and age (r = 0.464; *p* < 0.01), parity (r = 0.392; *p* < 0.05), number of live births (r = 0.373; *p* < 0.05), and the number of NSDs (r = 0.356; *p* < 0.05). The correlation coefficients showed that age was the parameter that most affected the degree of prolapse, followed by parity, number of live births, and number of NSDs.

The results of the Spearman’s rho correlation analysis for the relationship between POPQ Ap and POPQ Bp parameters with some demographic and obstetric characteristics of the patients in the study group are given in Table 4. The correlation analysis revealed statistically significant and positive correlations between the POPQ Ap and POPQ Bp parameters and age, parity, and number of live births. The results obtained for both POPQ Ap and POPQ Bp and the distribution of correlation coefficients were similar to those obtained for the degree of prolapse. According to the correlation coefficients, age was the parameter that affected POPQ Ap and POPQ Bp parameters the most, followed by parity, number of live births, and number of NSDs.

The results of the Spearman’s rho correlation analysis for the relationship between the parameters of muscle thickness and number of nerves per mm^2^ in fascia with some demographic and obstetric characteristics of the patients in the study group are given in Table 5. The correlation analysis revealed that age had a negative effect (r = −0.437; *p* < 0.05) and the CS number had a positive effect (r = 0.378; *p* < 0.05) on muscle thickness. There was no statistically significant correlation between demographic and birth characteristics with the parameters of muscle thickness and number of nerves per mm^2^ in fascia (*p* > 0.05).

## 4. Discussion

Means and ranges of change in gravida, parity, abortus, number of living children, number of NSDs, and duration of menopause were higher in the study group compared to the control group. POPQ Ap and POPQ Bp averages were statistically significantly higher in the control group, while muscle thickness and number of nerves per mm^2^ in fascia were significantly higher in the study group (*p* < 0.000). According to the correlation analysis results, there was a statistically significant and positive correlation between the degree of prolapse and POPQ Ap (r = 0.915; *p* < 0.01) and POPQ Bp (r = 0.912; *p* < 0.01). The correlation coefficient showed that the parameter that most affected the degree of prolapse was POPQ Ap. There was also a statistically significant and positive correlation between muscle thickness and the number of nerves per mm^2^ in the fascia (r = 0.618; *p* < 0.01). There was no statistically significant correlation between the degree of prolapse and muscle thickness (r = −0.026; *p* > 0.05) and the number of nerves per mm^2^ of fascia (r = −0.155; *p* > 0.05).

Vaginal wall prolapse is a condition classified as anterior, posterior, and apical compartments, characterized by muscle and fascia defects and causing numerous urinary, sexual, or bowel dysfunctions, especially incontinence.

In an imaging study of women with posterior vaginal prolapse, Luo et al. [28] reported the mean age of posterior vaginal prolapse in Caucasian women as 54.9 ± 8.7 years. In the same study, the mean age of the control group with similar symptoms was reported as 54.2 ± 8.9 years. In our study, the mean age of the patients was 49.87 ± 5.35 years in the control group and 61.13 ± 8.74 years in the study group. The age of the women with posterior vaginal wall prolapse was 60 years and above, consistent with the literature [3]. It is observed that the risk of POP development increases with age. In this case, age can be considered as an independent risk factor for rectocele.

Inal [17] reported that POP-Q Ap and POP-Q Bp scores were higher in the study group compared to the control group and the difference between the groups was statistically significant. Luo et al. [28] found the mean POP-Q Ap and POP-Q Bp to be 1.7 ± 0.8 in women with posterior vaginal prolapse. In the same study, the mean POP-Q Ap and POP-Q Bp of the control group were −1.7 ± 0.7, and the differences between the control and experimental groups were found to be statistically significant.

In our study, both POP-Q Ap and POP-Q Bp scores were statistically significantly higher in the study group compared to the control group (*p* < 0.05). In this respect, the results obtained in this study are compatible with both different prolapse results and posterior prolapse results. In other words, POP-Q Ap and POP-Q Bp averages are higher in women with prolapse compared to the control group.

Inal [17] reported that the number of nerves was statistically significantly lower in the prolapse group compared to the control group. In the same study, nerve diameter was statistically significantly higher in the control group than in the prolapse group. In our study, the mean of both muscle thickness and number of nerves per mm^2^ in fascia were statistically significantly higher in the control group than in the study group (*p* < 0.05). There was a statistically positive and significant correlation between muscle thickness and number of nerves in the fascia in the study group. Again, there was a statistically significant relationship between the degree of prolapse with POP-Q Ap and POP-Q Bp. Nonetheless, the relationships between the degree of prolapse with muscle thickness and the number of nerves per mm^2^ in the fascia were not statistically significant (*p* > 0.05).

In studies on the degree of prolapse, the risk factors for prolapse are reported to be the most important factor. Factors such as increasing age, number of NSDs, and number of live births increase the degree of prolapse [17,18,28]. In our study, the degree of prolapse was positively and significantly associated with age, parity, number of live births, and number of NSDs. POP-Q Ap and POP-Q Bp had similar associations with the degree of prolapse. Age had a negative and significant effect on muscle thickness, while the number of CS births was positively correlated with muscle thickness. That is, women who experienced more CSs had higher muscle thickness. It can be stated that CS birth prevents the reduction in muscle thickness.

İnal [17] reported a significant correlation between the number of nerves and age, NSD delivery, and postmenopausal period. All three variables negatively affect the number of nerves. In our study, the relationships between the number of nerves and age, NSD delivery, and postmenopausal period were not statistically significant (*p* > 0.05).

Studies on the innervation of the vaginal wall in patients with POP are inconclusive. The integrity of the vagina and supporting connective tissue is essential for normal pelvic floor function and the anatomy of the pelvic organs. Branches of the hypogastric plexus innervate the musculus levator ani and posterior vagina [29]. Denervation injury of the pelvic floor during labor may cause loss of vaginal support, leading to POP [30,31]. Several studies have evaluated anterior vaginal wall innervation in women with or without POP. Zhu et al. analyzed Protein Gene Product 9.5 (PGP 9.5) staining as a neuronal marker in peripheral nerves and ganglia in tissue [32]. They showed that the nerve fiber profile in the vaginal epithelium and subepithelium was significantly lower in women with stress urinary incontinence and POP compared to the control group. Inal et al. measured the number and diameter of subepithelial nerve fibers in the anterior vaginal wall and observed that these nerve fibers decreased in women with anterior prolapse compared to women with normal vaginal support [17]. Kaplan et al. confirmed this by describing reduced neuronization in the vaginal wall in the POP group [18].

To date, only two studies have evaluated the innervation of the posterior vaginal wall in women with or without POP. Boreham et al. [21] analyzed glial cells and astrocytes using antibodies against S100 and found that nerve fibers were fewer and smaller in the vaginal muscular layers of women in the POP group. Altman et al. reached the opposite conclusion by detecting increased nerve fiber density in the subepithelium of the rectovaginal wall in patients with posterior vaginal wall prolapse using PGP 9.5 antibodies. They suggested that neuronal regeneration following nerve trauma may be involved in the pathogenesis of pelvic floor disorders [33]. The discrepancy between these results can also be explained by the difference in the method of tissue sample collection, localization, and sensitivity of these two neuronal markers. Our study has some limitations. The first of these is that the average age of the groups we included in our study could not be matched despite all our efforts. This age difference may affect the results of the study. Conducting the study in age-matched groups may be the subject of other studies. In addition, our study was conducted with a limited number of women. Although the number of participants was statistically sufficient and significant, our study needs to be confirmed in larger groups.

Our study has the advantage of being a planned prospective study evaluating the innervation of the posterior vaginal wall in the literature, and the small number of cases is a limitation of our study.

## 5. Conclusions

Considering the results obtained in this study and the results reported in the literature together, it is observed that prolapse statistically significantly decreases the number of nerves in women. Similarly, it is reasonable to make the same interpretation for the degree of prolapse. Decreased innervation may lead to decreased smooth muscle thickness, although it is not very similar in structure to skeletal muscle. Further experiments with in vivo and in vitro models are necessary to clarify the cause-and-effect relationship between denervation and vaginal smooth muscle morphology.

## Figures and Tables

**Table 1:** Median of prolapse, POPQ Ap, POPQ Bp, muscle thickness, and number of nerves per mm^2^ in the fascia of the study and control groups and difference analysis results.

	Control (*n* = 31)	Study (*n* = 31)	*p*
	Median ± SD	Median ± SD	
Prolapsus degree	- -	3.23 ± 0.62	<0.05
POPQ Ap score	−2.68 ± 0.54	1.90 ± 1.04	0.000 ^a^
POPQ Bp score	−2.68 ± 0.54	5.26 ± 1.97	0.000 ^a^
muscle thickness (mm)	833.23 ± 145.09	443.74 ± 123.55	0.000 ^b^
number of nerves per mm^2^ in fascia (n)	10.67 ± 2.44	4.05 ± 1.52	0.000 ^b^

^a^. Mann–Whitney U Test, ^b^. Independent Sample T-Test, SD: Standard Deviation.

**Table 2:** Pearson’s correlation analysis results for the relationship between prolapse, POPQ Ap, POPQ Bp, muscle thickness, and the number of nerves per mm2 in the fascia in the study group.

	Prolapse Level	POPQ Ap	POPQ Bp	Muscle Thickness
POPQ Ap score	0.915 *	1		
POPQ Bp score	0.912 *	0.873 *	1	
muscle thickness (mm)	−0.026	−0.050	−0.185	1
the number of nerves per mm^2^ in the fascia (n)	−0.155	−0.209	−0.242	0.618 *

* *p* < 0.01.

**Table 3:** Correlation analysis results regarding the relationship between the degree of prolapse and some demographic and birth characteristics of the patients in the study group.

	r	*p*
Age (years)	0.464	0.008
Height (cm)	−0.067	0.720
Weight (kg)	0.013	0.945
BMI (kg/m^2^)	0.129	0.490
Smoke (*n*)	−0.298	0.110
Operation history (*n*)	0.030	0.875
Chronic disease (*n*)	0.032	0.866
Gravidity (*n*)	0.204	0.271
Parity (*n*)	0.392	0.029
Abortion (*n*)	−0.111	0.552
Vaginal birth (*n*)	0.356	0.050
Cesarean birth (*n*)	0.049	0.795
Duration of menopause (years)	0.320	0.127

**Table 4:** The relationship between the POPQ Ap and POPQ Bp parameters and some demographic and birth characteristics of the patients in the study group.

	POPQ Ap		POPQ Bp	
	r	*p*	r	*p*
Age (years)	0.475	0.007	0.536	0.002
Height (cm)	0.017	0.928	0.052	0.780
Weight (kg)	0.017	0.926	0.069	0.713
BMI (kg/m^2^)	0.102	0.586	0.138	0.460
Smoke (n)	−0.303	0.103	−0.215	0.254
Operation history	0.004	0.983	−0.064	0.732
Chronic disease (*n*)	0.004	0.982	−0.032	0.863
Gravidity (*n*)	0.172	0.355	0.275	0.134
Parity (*n*)	0.416	0.020	0.495	0.005
Abortion (*n*)	−0.203	0.272	−0.152	0.415
Vaginal birth (*n*)	0.373	0.038	0.457	0.010
Cesarian birth (*n*)	0.006	0.973	−0.043	0.817
Duration of menopause (years)	0.305	0.147	0.398	0.054

Spearman’s rho correlation analysis.

**Table 5:** The relationship between muscle thickness and nerve number per mm^2^ parameters in the fascia and some demographic and birth characteristics of the patients in the study group.

	Muscle Thickness		Nerve Number per mm^2^ Parameters in the Fascia	
	r	*p*	r	*p*
Age (years)	−0.437	0.014	−0.289	0.114
Height (cm)	−0.181	0.331	0.080	0.669
Weight (kg)	−0.159	0.394	0.024	0.897
BMI (kg/m^2^)	−0.107	0.565	−0.055	0.770
Smoke (*n*)	−0.006	0.973	0.122	0.521
Operation history (*n*)	0.185	0.319	−0.104	0.579
Chronic disease (*n*)	−0.064	0.734	−0.048	0.799
Gravidity (*n*)	−0.099	0.598	−0.078	0.677
Parity (*n*)	−0.146	0.432	−0.212	0.252
Abortion (*n*)	−0.059	0.752	0.125	0.505
Vaginal birth (*n*)	−0.228	0.217	−0.291	0.112
Cesarian birth (*n*)	0.378	0.036	0.342	0.060
Duration of menopause (years)	−0.391	0.059	−0.251	0.236

Spearman’s rho correlation analysis.

## Data Availability

The datasets used and/or analyzed during the current study are available from the corresponding author on reasonable request.
